# Enabling Fast and Accurate Record Linkage of Large-Scale Health-Related Administrative Databases Through a DNA-Encoded Approach.

**DOI:** 10.23889/ijpds.v7i3.1774

**Published:** 2022-08-25

**Authors:** José Araújo, Juan Silva, André Costa-Martins, Vanderson Sampaio, Daniel Castro, Robson Souza, Jeevan Giddaluru, Pablo Ramos, Robespierre Pita, Maurício Barreto, Manoel Netto, Helder Nakaya

**Affiliations:** 1 Universidade de São Paulo; 2 Fundação de Medicina Tropical Dr. Heitor Vieira Dourado; 3 Fundação de Vigilância em Saúde do Amazonas; 4 Oswaldo Cruz Foundation

## Background

Public health research frequently requires the integration of information from different data sources. However, errors in the records and the high computational costs involved make linking large administrative databases using record linkage (RL) methodologies a major challenge. We present Tucuxi-BLAST, a versatile tool for probabilistic RL that utilizes a DNA-encoded approach to encrypt, analyze and link massive administrative databases.

## Materials and Methods

Tucuxi-BLAST encodes the identification records into DNA. BLASTn algorithm is then used to align the sequences between databases. We tested and benchmarked on a simulated database containing records for 300 million individuals and also on four large administrative databases containing real data on Brazilian patients.

## Results

Our method was able to overcome misspellings and typographical errors in administrative databases. In processing the RL of the largest simulated dataset (200k records), the state-of-the art method took 5 days and 7 hours to perform the RL, while Tucuxi-BLAST only took 23 hours. When compared with five existing RL tools applied to a gold-standard dataset from real health-related databases, Tucuxi-BLAST had the highest accuracy and speed.

## Discussion

By repurposing genomic tools, researchers are able to perform subject tracing across multiple large epidemiological databases using a regular laptop.

## Conclusion

Tucuxi-BLAST can improve data-driven medical research and provide a fast and accurate way to link individual information across several administrative databases.

